# A Prospective Comparative Study Between Stapler and Handsewn Gastrointestinal Anastomosis

**DOI:** 10.7759/cureus.104423

**Published:** 2026-02-27

**Authors:** Gollapudi Sai Viswanth, Dayanand Biradar, Mallikarjun B Patil, Vikram Sindagikar, Anand Suntan, Veena Korishetty

**Affiliations:** 1 General Surgery, Shri B. M. Patil Medical College, Hospital and Research Centre, BLDE (Deemed to be University), Vijayapura, IND

**Keywords:** gastrointestinal anastomosis, handsewn anastomosis, operative time, postoperative outcomes, stapled anastomosis

## Abstract

Introduction: Gastrointestinal (GI) anastomosis is a critical step in bowel surgery, and the choice between stapled and handsewn techniques remains debated. Differences in operative efficiency and recovery have been reported, but complication rates appear comparable. This study compared stapled and handsewn GI anastomosis with respect to operative time, postoperative recovery, and complications.

Materials and methods: A prospective observational comparative study was conducted over two years in a tertiary care center. A total of 52 patients undergoing GI anastomosis were included, with 26 in the handsewn group and 26 in the stapler group. Technique selection was surgeon-directed. Baseline demographic and clinical variables were recorded. Primary outcomes included anastomosis time, time to oral feeding, and hospital stay. Secondary outcomes included anastomotic leak, surgical site infection (SSI), wound dehiscence, morbidity, and mortality. Continuous variables were analyzed using an independent t-test and categorical variables using a Chi-square test, with p < 0.05 considered significant.

Results: Baseline characteristics were comparable between groups. Anastomosis configuration differed significantly, with side-to-side performed in 26 (100%) stapled versus five (19.2%) handsewn cases (p < 0.001). Anastomotic leak occurred in two (7.7%) handsewn and three (11.5%) stapled cases (p = 0.638). SSI was seen in eight (30.8%) versus six (23.1%) (p = 0.532), and wound dehiscence in three (11.5%) versus zero (0%) (p = 0.074). The mean anastomosis time was significantly lower with stapling (5.81 ± 4.21 vs 25.62 ± 5.29 minutes, p < 0.001). Oral feeding was started earlier (2.31 ± 0.74 vs 4.27 ± 0.67 days, p < 0.001), and hospital stay was shorter (9.50 ± 2.18 vs 12.38 ± 2.12 days, p < 0.001). No mortality occurred.

Conclusion: Stapled anastomosis significantly improves operative efficiency and recovery while maintaining comparable complication rates.

## Introduction

Gastrointestinal (GI) surgery encompasses operative procedures involving the esophagus, stomach, small and large intestines, liver, pancreas, and gallbladder. These operations are performed for a wide range of conditions, including malignancies (such as colorectal, gastric, and pancreatic cancers), benign tumors, inflammatory bowel disease, mechanical obstruction, ischemia, trauma, and perforation [1]. Advances in surgical techniques, anesthesia, and perioperative care have markedly improved patient safety and outcomes. It is estimated that millions of GI surgeries are performed worldwide each year, a substantial proportion of which involve colorectal and small bowel resections [1,2].

Many GI operations involve resection of diseased bowel followed by restoration of intestinal continuity through an anastomosis [2,3]. The integrity of the anastomosis is a critical determinant of postoperative outcome, as failure can lead to serious complications such as anastomotic leak, hemorrhage, intra-abdominal abscess, peritonitis, sepsis, prolonged hospitalization, and increased mortality [4]. Therefore, construction of a tension-free, well-vascularized, and mechanically secure anastomosis remains one of the most important and technically demanding steps in GI surgery [5].

The technique of intestinal anastomosis has evolved considerably over time, from early handsewn methods described by Sushruta around 600 BC to the use of modern mechanical stapling devices [6,7]. Traditionally, handsewn anastomosis has been performed using layered suturing techniques with absorbable and/or nonabsorbable sutures [6,7]. Although versatile and cost-effective, handsewn anastomosis is time-consuming, technically demanding, and highly dependent on surgeon expertise [8]. Technological advances have resulted in widespread adoption of stapling devices, particularly in anatomically restricted fields and minimally invasive surgery, where manual suturing may be challenging [9].

Stapled anastomosis has been associated in several studies with reduced operative time, earlier return of bowel function, and shorter hospital stay [10,11]. However, its use is limited by higher cost, dependence on specialized equipment, and potential complications such as anastomotic leak, bleeding, stricture formation, and staple-line ischemia [12]. Consequently, in many resource-limited settings, handsewn anastomosis continues to play an essential role.

Despite widespread use of both techniques, the relative superiority of stapled versus handsewn anastomosis remains debated. Previous studies by Banurekha et al. and Boobalan et al. have reported comparable complication rates between stapled and handsewn methods, despite improved operative efficiency with staplers [13,14]. However, many available studies include heterogeneous operative sites and variable anastomotic configurations, and several are retrospective in design, which may limit causal interpretation. Furthermore, differences in anastomotic geometry (e.g., end-to-end versus side-to-side) may independently influence luminal diameter, perfusion, and postoperative recovery, thereby acting as potential confounders in technique-based comparisons.

Given these considerations and the continued clinical relevance of technique selection in diverse practice settings, a prospective observational comparison was undertaken. The primary objective of this study was to evaluate the association between anastomotic technique and operative efficiency, specifically anastomosis construction time. Secondary objectives included comparison of postoperative recovery parameters (time to resumption of oral feeding and duration of hospital stay) and short-term safety outcomes (anastomotic leak, surgical site infection (SSI), wound dehiscence, morbidity, and mortality).

## Materials and methods

This prospective observational comparative study was conducted in the Department of General Surgery at Shri B. M. Patil Medical College Hospital and Research Centre, Vijayapura, affiliated with BLDE (Deemed to be University), over a period of two years from January 2024 to December 2025. Institutional Ethics Committee approval (BLDE(DU)/IEC-SBMPMC/096/2023-24) was obtained prior to study initiation, and written informed consent was obtained from all participants. All patients were treated according to standard institutional surgical and perioperative protocols, and no change in clinical management was made for study purposes. The study was observational in nature, and no randomization or allocation concealment was performed.

The study population consisted of consecutive patients admitted under the Department of General Surgery who underwent GI anastomosis as part of elective or emergency surgical procedures during the study period. The choice of anastomotic technique (stapled or handsewn) was made by the operating surgeon based on clinical judgment, anatomical considerations, and resource availability. This surgeon-directed allocation may introduce selection bias and was not influenced by the study protocol. Eligible procedures included esophagogastric anastomosis, gastrojejunostomy, small bowel-small bowel anastomosis, small bowel-large bowel anastomosis, and large bowel-large bowel anastomosis. Both elective and emergency GI surgeries were included. Patients who underwent diversion procedures without restoration of intestinal continuity were excluded from the study.

Handsewn anastomoses were performed using a conventional double-layer technique consisting of an inner continuous absorbable suture layer and an outer interrupted seromuscular nonabsorbable layer. Stapled anastomoses were constructed primarily using linear cutting staplers for side-to-side configurations. All procedures were performed or supervised by consultant surgeons. Perioperative care followed standard institutional protocols and did not involve a formal enhanced recovery after surgery (ERAS) pathway.

Sample size estimation was based on previously published data by Banurekha et al., which reported a mean ± standard deviation of duration of procedure of 3 ± 0.433 in the handsewn group and 2.25 ± 0.758 in the stapled group [13]. Using these values, the required sample size for comparison of two independent means was calculated using the standard formula N = 2[(Zα + Zβ) × S / d]² for two-group comparisons. A two-sided significance level (α) of 5% and statistical power of 95% (1-β) were assumed, corresponding to Zα = 1.96 and Zβ = 1.64. Based on the pooled standard deviation and the expected clinically meaningful difference between groups, the minimum required sample size was 26 patients per group, resulting in a total sample size of 52.

Data were collected prospectively using a structured case record proforma. Preoperative variables included age, sex, diagnosis, comorbidities, indication for surgery, and relevant laboratory and radiological findings. Operative details recorded included urgency of surgery (elective or emergency), type of procedure, anatomical site, type of anastomosis, and technique used (stapled or handsewn). Anastomotic construction time was measured in minutes using a standard operating room timer, defined as the interval from the beginning of bowel approximation to completion of suturing or stapling.

Postoperative monitoring was performed daily until discharge. Time to return of bowel function was defined clinically by the passage of flatus or stool. Time to resumption of oral feeding was recorded as the number of postoperative days from surgery to initiation of oral intake. Criteria for initiation of oral feeding were based on clinical assessment of bowel activity and hemodynamic stability at the discretion of the treating surgical team. Anastomotic leak was defined as a defect in the integrity of the intestinal anastomosis leading to communication between intra- and extraluminal compartments, diagnosed based on clinical features (such as fever, tachycardia, abdominal pain, peritonitis), radiological evidence on contrast imaging, or the presence of feculent or enteric discharge from drains or wound sites, consistent with standard surgical definitions [15].

SSI was defined according to the Centers for Disease Control and Prevention (CDC) criteria as an infection occurring within 30 days after surgery (or within 90 days if an implant is in place) involving the incision or deep tissues at the operative site, and characterized by purulent discharge, positive culture from the wound, deliberate opening of the incision by the surgeon with signs of infection, or diagnosis of SSI by the treating surgeon [16]. Wound dehiscence was defined as partial or complete separation of the layers of a surgical wound after closure [17]. Need for reoperation and in-hospital mortality were also recorded. Total hospital stay was calculated in days from the date of surgery to the date of discharge.

All data were entered into MS Excel (Microsoft Corporation, Redmond, Washington, United States) and analyzed using IBM SPSS Statistics for Windows, Version 26 (Released 2018; IBM Corp., Armonk, New York, United States). Continuous variables were expressed as mean ± standard deviation and analyzed using an independent t-test. Categorical variables were presented as frequencies and percentages and analyzed using the Chi-square test. A p-value < 0.05 was considered statistically significant, and all statistical tests were two-tailed.

## Results

The study included 52 patients, with equal distribution between the handsewn and stapler groups. Age distribution was comparable across groups, with the highest representation in the 41-50 years category in both handsewn (7, 26.9%) and stapler (7, 26.9%) groups, followed by 31-40 years with six (23.1%) and three (11.5%), respectively. Other age bands were similarly balanced, and no significant difference in age distribution was observed (p = 0.893). Female patients constituted 14 (53.8%) in the handsewn group and 10 (38.5%) in the stapler group, while males accounted for 12 (46.2%) and 16 (61.5%), respectively (p = 0.266). Most patients belonged to ASA grade III, with 13 (50.0%) in the handsewn and 10 (38.5%) in the stapler group, followed by ASA II with 10 (38.5%) and nine (34.6%); overall, ASA distribution did not differ significantly (p = 0.486). No statistically significant baseline demographic or preoperative clinical differences were detected between groups (Table 1).

**Table 1:** Baseline demographic and clinical characteristics by anastomotic technique ASA: American Society of Anesthesiologists This table presents the comparison of baseline demographic and preoperative clinical characteristics between patients undergoing handsewn and stapled anastomosis. Variables include age distribution (categorized into six age groups), sex, and ASA grade. Data are expressed as frequency and percentage (n (%)). The chi-square (χ²) test was applied to assess differences between groups, with corresponding p-values reported

Variable	Category	Handsewn n (%)	Stapler n (%)	χ² value	p-value
Age (years)	20-30	5 (19.2)	7 (26.9)	1.667	0.893
	31-40	6 (23.1)	3 (11.5)		
	41-50	7 (26.9)	7 (26.9)		
	51-60	5 (19.2)	5 (19.2)		
	61–70	2 (7.7)	2 (7.7)		
	71-80	1 (3.8)	2 (7.7)		
Sex	Female	14 (53.8)	10 (38.5)	1.238	0.266
	Male	12 (46.2)	16 (61.5)		
ASA grade	I	2 (7.7)	3 (11.5)	2.444	0.486
	II	10 (38.5)	9 (34.6)		
	III	13 (50.0)	10 (38.5)		
	IV	1 (3.8)	4 (15.4)		

Ischemic or gangrenous bowel was the most common indication for surgery in both groups, seen in 13 (50.0%) handsewn and 11 (42.3%) stapler cases. Malignancy accounted for four (15.4%) in the handsewn and seven (26.9%) in the stapler group. Perforation was noted in five (19.2%) and three (11.5%), while trauma-related indications were present in two (7.7%) and three(11.5%), respectively. Fistula or miscellaneous causes contributed two (7.7%) cases in each group. The distribution of diagnoses was not significantly different between techniques (p = 0.437) (Table 2).

**Table 2:** Comparison of diagnosis by anastomotic technique This table illustrates the distribution of surgical indications among patients in the hand-sewn and stapler groups. Diagnoses include malignancy, ischemic/gangrenous bowel, perforation, trauma (RTA), and fistula/miscellaneous causes. Values are presented as number and percentage (n (%)). The chi-square (χ²) test was used to compare diagnostic categories between groups, and p-values are provided to determine statistical significance

Diagnosis	Handsewn n (%)	Stapler n (%)	χ²	p-value
Malignancy	4 (15.4)	7 (26.9)	3.804	0.437
Ischemic/gangrenous	13 (50.0)	11 (42.3)		
Perforation	5 (19.2)	3 (11.5)		
Trauma (RTA)	2 (7.7)	3 (11.5)		
Fistula/misc	2 (7.7)	2 (7.7)		

Marked differences were observed in anastomotic configuration. End-to-end anastomosis was performed in 20 (76.9%) handsewn cases and none in the stapler group, whereas side-to-side anastomosis was used in five (19.2%) handsewn and 26 (100%) stapler cases (p < 0.001). Regarding the site, ileoileal anastomosis was more frequent in the handsewn group with 15 (57.7%) compared to five (19.2%) in the stapler group, while ileotransverse and jejunojejunal sites were more common with stapling at 14 (53.8%) and 6(23.1%) versus five (19.2%) and two (7.7%), respectively. Esophagojejunostomy was performed only in handsewn cases (3, 11.5%). Site distribution differed significantly between groups (p = 0.013) (Table 3).

**Table 3:** Anastomosis characteristics This table summarizes the operative characteristics of anastomosis in both groups, including the type of anastomosis (end-to-end, end-to-side, side-to-side) and the site of anastomosis (esophagojejunostomy, ileoileal, ileotransverse, jejunoileal, jejunojejunal). Data are presented as frequency and percentage (n (%)). Intergroup comparisons were performed using the chi-square (χ²) test, and corresponding p-values are reported

Variable	Category	Handsewn n (%)	Stapler n (%)	χ² value	p-value
Type of anastomosis	End-to-end	20 (76.9)	0 (0.0)	32.732	<0.001
	End-to-side	1 (3.8)	0 (0.0)		
	Side-to-side	5 (19.2)	26 (100)		
Site of anastomosis	Esophagojejunostomy	3 (11.5)	0 (0.0)	14.361	0.013
	Ileoileal	15 (57.7)	5 (19.2)		
	Ileotransverse	5 (19.2)	14 (53.8)		
	Jejunoileal	1 (3.8)	1 (3.8)		
	Jejunojejunal	2 (7.7)	6 (23.1)		

Postoperative complication rates did not demonstrate statistically significant differences between techniques. Anastomotic leak occurred in two (7.7%) handsewn and three (11.5%) stapler cases (p = 0.638). SSI was recorded in eight (30.8%) and six (23.1%), respectively (p = 0.532). Wound dehiscence was observed only in the handsewn group with three (11.5%) cases and none in the stapler group, approaching but not reaching statistical significance (p = 0.074). Overall, no complication parameter showed a statistically significant difference between groups. No patients died during the study period, with no mortality observed in both the handsewn and stapled anastomosis groups (Table 4).

**Table 4:** Postoperative complications by anastomotic technique NA: not applicable This table compares postoperative outcomes between handsewn and stapled anastomosis groups. Complications assessed include anastomotic leak, surgical site infection, wound dehiscence, and mortality. Results are expressed as numbers and percentages (n (%)). The chi-square (χ²) test was used for categorical comparisons, and p-values are provided. Mortality was NA for statistical testing due to the absence of events in both groups

Outcome	Handsewn n (%)	Stapler n (%)	χ²	p-value
Anastomotic leak	2 (7.7)	3 (11.5)	0.221	0.638
Surgical site infection	8 (30.8)	6 (23.1)	0.391	0.532
Wound dehiscence	3 (11.5)	0 (0.0)	3.184	0.074
Mortality	0 (0.0)	0 (0.0)	NA	NA

Operative and recovery parameters showed significant differences favoring stapled anastomosis. Mean anastomosis time was substantially shorter in the stapler group compared with the handsewn group (p < 0.001). Time to initiation of oral feeding was earlier in the stapler group (p < 0.001). Duration of hospital stay was also shorter with stapled anastomosis (p < 0.001). All continuous outcome measures demonstrated statistically significant improvement with the stapling technique (Table 5).

**Table 5:** Comparison of recovery and operative time parameters between groups SD: standard deviation; POD: postoperative day This table presents the comparison of continuous operative and recovery parameters between the handsewn and stapled anastomosis groups. Variables include anastomosis time (minutes), POD of initiation of oral feeding, and duration of hospital stay (days). Data are expressed as mean ± SD. Intergroup comparisons were performed using the independent samples t-test, with corresponding t-values and p-values reported

Variable	Handsewn (mean ± SD)	Stapler (mean ± SD)	t-value	p-value
Anastomosis time (minutes)	25.62 ± 5.29	5.81 ± 4.21	15.21	<0.001
Oral feeding day (POD)	4.27 ± 0.67	2.31 ± 0.74	10.05	<0.001
Hospital stay (days)	12.38 ± 2.12	9.50 ± 2.18	4.79	<0.001

## Discussion

GI anastomosis remains a central step in bowel reconstruction, and both handsewn and stapled techniques continue to be widely used. Across comparative studies, baseline demographic and risk profiles are generally similar between technique groups, supporting fair outcome comparisons. Mehta et al. reported male predominance of 31 (62%), with the 41-50 year age group most commonly represented [18]. Boobalan et al. observed male predominance of 29 (58%) among 50 patients with comparable distribution between techniques [14]. Bhattacharjee et al. documented closely matched mean ages between handsewn and stapled groups, and Sarkar et al. found no significant difference in age or sex distribution between cohorts (p > 0.7) [19,20].

Anastomotic configuration and site selection often differ by technique, with staplers more commonly used for side-to-side reconstruction and handsewn methods for end-to-end anastomoses. Banurekha et al. and Boobalan et al. reported higher stapler use in ileocolic and side-to-side reconstructions, whereas handsewn methods were more frequent in end-to-end joins [13,14]. Such configuration differences are important because anastomotic geometry may independently influence functional recovery and luminal patency and therefore represent a potential confounder when comparing techniques across heterogeneous operative sites.

Reduced operative and anastomosis construction time with stapled techniques is one of the most consistent findings across studies. Mehta et al. reported faster anastomosis construction with staplers (11.80 ± 2.44 vs 17.80 ± 2.53 minutes, p = 0.001) [18]. Boobalan et al. demonstrated shorter mean operative duration with staplers (127.88 ± 22.23 vs 158.38 ± 26.86 minutes, p < 0.001) [14]. Banurekha et al. showed reduced operative time across multiple procedures, including subtotal gastrectomy (1.93 vs 2.71 hours, p = 0.012) and right hemicolectomy (2.0 vs 3.0 hours, p = 0.018) [13]. Sarkar et al. similarly reported shorter operative time with stapling (144.8 vs 166.2 minutes, p < 0.0001) [20]. Across reports, the numerical differences consistently support stapling as the more time-efficient technique.

With respect to safety outcomes, most studies show broadly comparable complication rates between techniques. Mehta et al. reported anastomotic leak in four (16%) handsewn versus two (8%) stapled cases (p = 0.384) [18]. Banurekha et al. observed a leak in eight (28.6%) patients in the handsewn group versus three (13.6%) in the stapled group, approaching significance (p = 0.074), with mortality occurring in three (10.7%) handsewn and zero (0%) stapled cases (p = 0.038) [13]. Sarkar et al. documented identical leak rates of 1(1.47%) in each group (p = 1.000) [20]. Bhattacharjee DS et al. reported leak rates of four (13.33%) versus three (10%) in handsewn and stapled groups, respectively [19]. Kshirsagar et al. demonstrated elective-case leak in four (16%) handsewn versus zero (0%) stapled patients (p = 0.04), but no significant difference in emergency surgery (p = 0.1) [11]. Boobalan et al. noted higher overall morbidity in handsewn groups without consistent statistical significance [14]. These numerical findings collectively indicate near-equivalent safety profiles.

Recovery outcomes in several series favor stapled reconstruction, particularly for earlier feeding and shorter hospitalization. Banurekha et al. reported earlier oral intake (2.25 vs 3.14 days, p = 0.004) and reduced hospital stay (8.0 vs 10.14 days, p = 0.001) with staplers [13]. Boobalan et al. found shorter hospital stay with stapled anastomosis (12.27 ± 2.07 vs 14.79 ± 1.41 days, p < 0.001) [14]. Bhattacharjee et al. observed earlier feeding (4.57 vs 4.8 days) and shorter stay (10.07 vs 10.67 days) with staplers, though without formal significance testing [19]. In contrast, Sarkar et al. found similar recovery timelines between techniques, with bowel activity around 95 hours in both groups and hospital stay near eight days (p > 0.7) [20]. Taken together, published numerical data suggest that stapled anastomosis often supports faster recovery, although the effect size varies with perioperative care pathways and case mix.

Strengths and limitations

The study has several notable strengths, including its prospective design, equal group sizes, and well-matched baseline demographic and clinical characteristics, which enhance internal validity and allow balanced comparison between stapled and hand-sewn techniques. Multiple outcome domains were evaluated, such as operative time, anastomotic configuration, postoperative complications, and recovery parameters, providing a comprehensive assessment rather than a single-endpoint comparison, and the findings were interpreted alongside published numerical data from prior studies to improve contextual relevance.

However, certain limitations should be acknowledged. The sample size was modest, which limits statistical power for less frequent outcomes such as anastomotic leak and mortality. The observational, nonrandomized design introduces potential selection and technique-allocation bias. The most critical limitation is the statistically significant imbalance in anastomotic configuration and site between groups, which represents a major confounding factor and may independently influence recovery outcomes. No multivariable adjustment or stratified subgroup analysis was performed due to sample size constraints. Heterogeneity in operative indications further limits interpretability. Long-term outcomes such as stricture formation and functional performance were not assessed. Although surgeon experience was comparable, inter-operator variability cannot be fully excluded. Cost-effectiveness analysis was not performed, which may be particularly relevant in resource-limited settings. These factors may affect generalizability across different institutions and healthcare systems.

## Conclusions

Stapled GI anastomosis was associated with improved operative efficiency and postoperative recovery parameters, including reduced anastomosis construction time, earlier initiation of oral feeding, and shorter hospital stay compared with handsewn techniques. No statistically significant differences were observed in anastomotic leak, SSI, wound dehiscence, overall morbidity, or mortality between the two techniques. Both techniques remain safe and effective when performed according to established surgical principles. Stapled anastomosis may be advantageous where operative time and faster recovery are priorities, whereas handsewn anastomosis remains a dependable and adaptable method, particularly when stapling devices are unavailable or unsuitable. The choice of technique should be individualized based on patient factors, intraoperative findings, and surgical judgment.

## References

[1] Küper MA, Eisner F, Königsrainer A, Glatzle J (2014). Laparoscopic surgery for benign and malign diseases of the digestive system: indications, limitations, and evidence. World J Gastroenterol.

[2] Kopecky KE, Monton O, Koti S (2026). Perioperative expectations of patients undergoing gastrointestinal surgery for cancer: a systematic review. Ann Surg Oncol.

[3] Newton C, Fichera A (2025). Anastomosis after bowel resection for Crohn's disease: state of the art review. Clin Colon Rectal Surg.

[4] Numaro YT, Kebede MA, Mariam SN (2025). Clinical anastomosis leakage and determinant factors among patients who underwent intestinal anastomosis in two Ethiopian tertiary hospitals. BMC Gastroenterol.

[5] Goulder F (2012). Bowel anastomoses: the theory, the practice and the evidence base. World J Gastrointest Surg.

[6] Yao L, Li C, Zhu X, Shao Y, Meng S, Shi L, Wang H (2016). An effective new intestinal anastomosis method. Med Sci Monit.

[7] Khoury GA, Waxman BP (1983). Large bowel anastomoses. I. The healing process and sutured anastomoses. A review. Br J Surg.

[8] Varela C, Nassr M, Razak A, Kim NK (2022). Double-layered hand-sewn anastomosis: a valuable resource for the colorectal surgeon. Ann Coloproctol.

[9] Chekan E, Whelan RL (2014). Surgical stapling device-tissue interactions: what surgeons need to know to improve patient outcomes. Med Devices (Auckl).

[10] Maatooq MA, Merdan I (2017). Comparative study between stapler and hand sewing in gastrointestinal anastomosis. Basrah J Surg.

[11] Kshirsagar VV, Mp H (2024). A comparative study of hand-sewn and stapled anastomosis in gastrointestinal surgeries. Cureus.

[12] Holzmacher JL, Luka S, Aziz M, Amdur RL, Agarwal S, Obias V (2017). The use of robotic and laparoscopic surgical stapling devices during minimally invasive colon and rectal surgery: a comparison. J Laparoendosc Adv Surg Tech A.

[13] Banurekha R, Sadashivam S, Sathyamoorthy K (2017). Hand sewn versus stapler anastomosis in elective gastro intestinal surgeries. Int Surg J.

[14] Boobalan M, Dharmarajan M, Udhayasuriyan R (2023). A comparative study between stapler and hand-sewn anastomosis in gastrointestinal surgeries. Int J Acad Med Pharm.

[15] Smith H, Brooks JE, Leaptrot D (2017). Health care-associated infections studies project: an American Journal of Infection Control and National Healthcare Safety Network data quality collaboration. Am J Infect Control.

[16] Borchardt RA, Tzizik D (2018). Update on surgical site infections: the new CDC guidelines. JAAPA.

[17] Lakshmi G, Ravimohan TR (2018). Post laparotomy abdominal wound dehisence-a study in tertiary care hospital. Int J Contemp Med Res.

[18] Mehta A, Sharma P, Pancholi M (2023). A retrospective comparative study on stapler verses hand sewn technique in gastrointestinal anastomosis of twenty-five cases each. Int Surg J.

[19] Bhattacharjee DS, Das PDB, Killing DD (2025). A competitive study of stapler bowel anastomosis and conventional hand sewn bowel anastomosis in regard to outcome and complications in a tertiary care hospital of upper assam. J Cardiovasc Dis Res.

[20] Sarkar AN, Deb PP, Naskar S (2024). A comparative study between stapler and hand sewn anastomosis in elective gastrointestinal surgery in a tertiary care centre. Sch J App Med Sci.

